# LAD: Layer-Wise Adaptive Distillation for BERT Model Compression

**DOI:** 10.3390/s23031483

**Published:** 2023-01-28

**Authors:** Ying-Jia Lin, Kuan-Yu Chen, Hung-Yu Kao

**Affiliations:** Department of Computer Science and Information Engineering, National Cheng Kung University, Tainan 70101, Taiwan

**Keywords:** model compression, knowledge distillation, BERT, text classification, natural language processing, deep learning

## Abstract

Recent advances with large-scale pre-trained language models (e.g., BERT) have brought significant potential to natural language processing. However, the large model size hinders their use in IoT and edge devices. Several studies have utilized task-specific knowledge distillation to compress the pre-trained language models. However, to reduce the number of layers in a large model, a sound strategy for distilling knowledge to a student model with fewer layers than the teacher model is lacking. In this work, we present **L**ayer-wise **A**daptive **D**istillation (LAD), a task-specific distillation framework that can be used to reduce the model size of BERT. We design an iterative aggregation mechanism with multiple gate blocks in LAD to adaptively distill layer-wise internal knowledge from the teacher model to the student model. The proposed method enables an effective knowledge transfer process for a student model, without skipping any teacher layers. The experimental results show that both the six-layer and four-layer LAD student models outperform previous task-specific distillation approaches during GLUE tasks.

## 1. Introduction

In recent years, large-scale pre-trained language models such as BERT [1], RoBERTa [2], XLNet [3], ELECTRA [4], and GPT [5] led to breakthroughs in natural language processing (NLP). Fine-tuning these pre-training frameworks with downstream tasks as a form of transfer learning has become the de facto standard in NLP. Recent research [6,7] also indicates that even larger models can lead to a better performance on downstream tasks, which has caused scientists to put more effort into building a much larger language model. Even though these growing trends of building large pre-trained language models have improved the performance of numerous NLP downstream tasks, these state-of-the-art models are too big to be deployed on computationally limited devices such as IoT or smartphones, which hampers the wide application of pre-trained language models. As a result, compressing large-sized pre-trained language models into moderate scales is an important issue.

Knowledge distillation [8,9] is a model compression technique that aims to transfer knowledge from a larger teacher model to a smaller student model, thus reducing the number of model parameters. Recently, there have been several breakthroughs [10,11,12,13] related to the compression of BERT models in the pre-training stage, which is also called task-agnostic distillation [13]. To prevent re-building a pre-trained language model, researchers [14,15] are seeking an alternative that can directly distill knowledge from a teacher model for a downstream task, such as task-specific distillation [13]. In this way, given a downstream task, the teacher is the BERT model that was fine-tuned on the task, and the goal of the student model is to mimic the outputs of the teacher during the given task. Different from the traditional knowledge distillation approach [8], Sun et al. [14] proposed Patient Knowledge Distillation (PKD). PKD allows for a student to effectively obtain the teacher model’s knowledge by minimizing the differences in the hidden states of each layer between the teacher model and the student model. Although PKD achieves comparable performances on various downstream tasks, the approach skips every two layers of the teacher model during distillation, which makes it a sub-optimal strategy for distilling layer-wise knowledge from the teacher model.

To fix this problem in PKD [14], instead of skipping some teacher layers, Passban et al. [15] proposed Attention-Based Layer Projection for Knowledge Distillation (ALP-KD) to optimize the student model with all layers in the teacher model. However, each layer in BERT [1] plays a role in the NLP pipeline [16]. The lower layers of BERT handle more local syntax, while the higher layers are in charge of complex semantics [16]. As a result, BERT’s sentence processing depends on these layer-by-layer sequential patterns [16]. In other words, the strategy of distilling higher layers of a teacher model to lower layers of a student model in ALP-KD [15] violates the nature of BERT.

**Figure 1:**
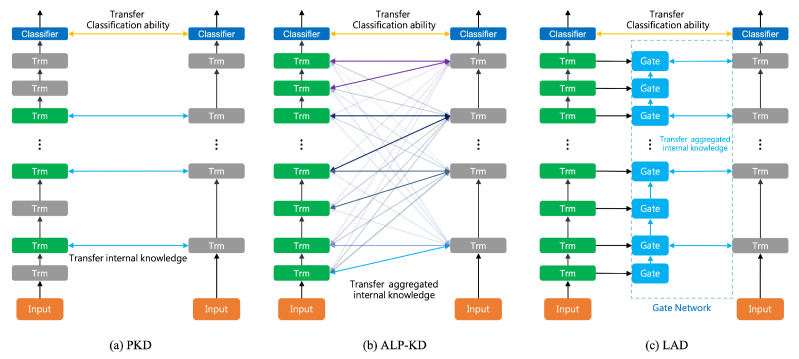
Differences in model structures of three distillation frameworks. Trm means a Transformer layer. In each subplot, the left shows the teacher model, and the right indicates the student model. (**a**) Patient knowledge distillation (PKD) [14]; (**b**) Attention-Based Layer Projection for Knowledge Distillation (ALP-KD) [15]; (**c**) Our proposed method.

To solve the problems related to both PKD [14] and ALP-KD [15] when improving model compression, in this work, we propose **L**ayer-wise **A**daptive **D**istillation (LAD). Inspired by the Highway Networks [17], we designed a Gate Network with multiple gate blocks in LAD. Our proposed LAD framework with a Gate Network is shown in Figure 1c. Each gate block is equipped with an adjustable weight matrix to adaptively determine the distillation ratio from multiple teacher layers to a single layer in the student model, which resolves the layer selection problem [14] that occurs when distilling knowledge from a teacher to a student. In addition, the LAD framework utilizes an iterative aggregation mechanism to retain the sequential patterns of processing text in BERT layers [16], which relieves the problem of exposing all of the teacher layers when training a student model [15]. We list our contributions as follows:

- We designed a novel task-specific distillation framework called Layer-wise Adaptive Distillation (LAD), which can train the student model without skipping any teacher layers for better model compression.
- The proposed method achieved competitive performances on several GLUE tasks and reduced the performance gap between the teacher and the student model.
- The proposed method can benefit task-specific distillation by retaining the sentence-processing nature of BERT [16].
- Our method can further be applied to IoT or edge devices to leverage the pre-trained language models for natural language applications.

## 2. Related Work

Recently, the compression of BERT in terms of knowledge distillation has attracted the attention of NLP researchers. DistillBERT [10] is a small pre-trained BERT model, which was trained by mimicking the output of masked language modeling from the teacher model. Its model size is 40% smaller (66M) than a BERT_BASE_ teacher (110M), and achieves a comparable performance on various downstream tasks. TinyBERT [11] and its variant BERT-EMD [18] further minimize the differences in self-attention distribution when distilled from a pre-trained teacher. MobileBERT [12] also re-designed the Transformer layer architecture to reduce the number of parameters in each Transformer layer. Although these approaches can successfully make large-sized models into smaller ones and gain comparable performances to the corresponding teacher model, the model and the training procedures both need to be re-formulated for the purpose of distilling knowledge from the teacher model to the student model. Therefore, in this work, we focus on exploring task-specific knowledge distillation methods.

The PKD [14] and ALP-KD [15] models are directly related to our study. PKD has two variants: PKD-Last and PKD-Skip. The former distills the knowledge of the last *p* layers in the teacher model to the student model; the latter only distills the knowledge from every *p* layer to the student model. Although PKD-Skip has a better model performance on the GLUE tasks, this method ignores the information in the skipped layers. To better distill the knowledge from the teacher model, ALP-KD [15] proposed an attention-based strategy to dynamically determine the distillation ratio from all layers in the teacher model. Nevertheless, according to their experiments, ALP-KD does not exhibit significant improvements in model performances. This circumstance may result from the distillation of higher teacher layers to lower student layers in ALP-KD, which violates the sentence-processing nature of BERT [16].

Differing from PKD [14] and ALP-KD [15], to more effectively distill knowledge from every layer in the teacher model, we leverage additional weight matrices in our Gate Network to help the student model adaptively learn the information in multiple teacher layers during training.

## 3. Materials and Methods

### 3.1. Internal Knowledge in Teacher Layers

Assume we have a labeled dataset $X={\left\{x_{i},y_{i}\right\}}_{i=1}^{K}$, where *K* is the number of samples in the dataset *X*. We denote the *i*-th input instance as $x_{i}$ and *i*-th output instance as $y_{i}$. Given input $x_{i}$ to our teacher model $f^{t}$, a sequence of hidden states $H_{i}^{t}$ is generated, as follows:

(1) $$\begin{array}{cccccccccc} h_{i,n}^{t}=f_{n}^{t}\left(x_{i};\theta_{n}^{t}\right), & & & & & & & & & \;n=1,\cdots ,N; \end{array}$$

(2) $$\begin{array}{cccccccccccccc} H_{i}^{t} & =\{h_{i,1}^{t},h_{i,2}^{t},\cdots ,h_{i,N}^{t}\} & & & & & & & & & & & & \end{array}$$

where $h_{i,n}^{t}\in R^{l\times d}$ is the output hidden states from *n*-th teacher layer given input $x_{i}$, *l* is the input sequence length and *d* is the hidden dimension of teacher model $f^{t}$. $f_{n}^{t}\left(x_{i};\theta_{n}^{t}\right)$ is the *n*-th layer of the teacher model with learnable parameters $\theta_{n}^{t}$, and *N* is the number of teacher layers. $H_{i}^{t}$ represents the internal knowledge of all teacher layers.

### 3.2. Gate Block

Consider the *n*-th layer of our teacher model $f^{t}$. For the purpose of explaination, we intentionally left the notation of *n* and denote teacher hidden state as *h*. Let $\hat{h}$ be the previous output of gate block *G*, where

(3) $$\begin{array}{c} G\left(h,\hat{h}\right)=\mathrm{LN}\left(\hat{h}\cdot T\left(h\right)+h\cdot \left(1-T\left(h\right)\right)\right). \end{array}$$

Here, LN is layer normalization [19], and T(h) is the *Transform gate*:

(4) $$\begin{array}{c} T(h)=\sigma (Wh+b), \end{array}$$

where σ is sigmoid function and W,b are learnable parameters. The output of the *Transform gate* T(h) is a tensor; each value in the tensor T(h) ranges from 0 to 1. Equation (3) can be viewed as a linear combination of its input, and *Transform gate* T(h) can be viewed as the weighted coefficients of Equation (3). Later on, we will replace *h* with $h_{i,n}^{t}$ to specifically denote the layer information of a given instance $x_{i}$.

### 3.3. Iterative Aggregation Mechanism

To retain the sentence processing properties of BERT [16], we applied the gate block defined as above. We designed an iterative algorithm to aggregate information in our framework. The hidden state ${\hat{h}}_{i,n}$ of *n*-th gate block $G_{n}$ with respect to input $x_{i}$ is defined as:

(5) $${\hat{h}}_{i,n}=\left\{\begin{array}{cc} G_{n}\left(h_{i,n}^{t},0\right) & \mathrm{if}\;n=1\\ G_{n}\left(h_{i,n}^{t},{\hat{h}}_{i,n-1}\right) & \mathrm{if}\;n=2,\cdots ,N \end{array}\right.$$

Here, $h_{i,n}^{t}$ is the hidden state of *n*-th teacher layer given input $x_{i}$. ${\hat{h}}_{i,n-1}$ is the hidden state with aggregated information from previous teacher layers. With the operation in Equation (5), the aggregated hidden states ${\hat{h}}_{i,n-1}$ can be iteratively passed layer by layer. We collectively denoted all the aggregated hidden states as ${\hat{H}}_{i}$:

(6) $$\begin{array}{c} {\hat{H}}_{i}=\left\{{\hat{h}}_{i,1},{\hat{h}}_{i,2},\cdots ,{\hat{h}}_{i,N}\right\} \end{array}$$

Taking a six-layer student model as an example, we can obtain a sequence of hidden states $H_{i}^{s}$ given input $x_{i}$:

(7) $$\begin{array}{cccccccc} h_{i,m}^{s}=f_{m}^{s}\left(x_{i};\theta_{m}^{s}\right), & & & & & & & m=1,\cdots ,6; \end{array}$$

(8) $$\begin{array}{ccccccccccc} H_{i}^{s}=\{h_{i,1}^{s},h_{i,2}^{s},\cdots ,h_{i,6}^{s}\}, & & & & & & & & & & \end{array}$$

where $f_{m}^{s}$ represents the *m*-th layer of the student model $f^{s}$ with learnable parameters $\theta_{m}^{s}$, and $h_{i,m}^{s}\in R^{l\times d}$ is the output hidden state from the *m*-th student layer. Then, we can take every *p*’s hidden states from ${\hat{H}}_{i}$, defined in Equation (6), and restrict p=N/M, where *M* is the number of layers in $f^{s}$. In the case of distilling knowledge from a 12-layer teacher to a 6-layer student model, we have p=12/6=2, which means we learn information from every even teacher layer:

(9) $$\begin{array}{c} {\tilde{H}}_{i}=\left\{{\hat{h}}_{i,2},{\hat{h}}_{i,4},{\hat{h}}_{i,6},{\hat{h}}_{i,8},{\hat{h}}_{i,10},{\hat{h}}_{i,12}\right\}. \end{array}$$

We then optimize our student model $f^{s}$ by minimizing the mean square error between $H_{i}^{s}$ and ${\tilde{H}}_{i}$:

(10) $$\begin{array}{cc} L_{\mathrm{hidden}} & =\sum_{i=1}^{K}\sum_{m=1}^{M}{\left(h_{i,m}^{s}-{\hat{h}}_{i,mp}\right)}^{2}. \end{array}$$

Note again that *K* is the number of samples in our dataset.

### 3.4. Learn Predictions from the Teacher

In addition to leveraging the aggregated internal knowledge, we encourage the student model to learn predictions from the teacher. We extract the soft labels ${\tilde{y}}_{i}^{t}$ for each input instance $x_{i}$ from a teacher model:

(11) $$\begin{array}{cccc} \;z_{i}^{t}=f^{t}\left(x_{i};\theta_{t}\right) & & & \end{array}$$

(12) $$\begin{array}{c} {\tilde{y}}_{i}^{t}=\mathrm{softmax}(z_{i}^{t}/\tau ) \end{array}$$

where $z_{i}^{t}$ is the output logits of the teacher model, and τ is the softmax temperature [8]. We can also obtain the output probability ${\tilde{y}}_{i}^{s}$ of a student model for any given instance $x_{i}$ in a similar way:

(13) $$\begin{array}{cccc} z_{i}^{s}=f^{s}\left(x_{i};\theta_{s}\right) & & & \end{array}$$

(14) $$\begin{array}{cc} {\tilde{y}}_{i}^{s} & =\mathrm{softmax}(z_{i}^{s}/\tau ) \end{array}$$

where $z_{i}^{s}$ is the output logits of the student model, and the softmax temperature is also denoted as τ. Then, we calculate the KL-divergence of ${\tilde{y}}_{i}^{t}$ and ${\tilde{y}}_{i}^{s}$ to measure the distance of probability distributions between the soft labels from the teacher model and the output from the student model:

(15) $$\begin{array}{c} L_{\mathrm{soft}}=D_{KL}({\tilde{y}}^{t}\left|\right|{\tilde{y}}^{s})=\sum_{i=1}^{K}{\tilde{y}}_{i}^{t}\log\left(\frac{{\tilde{y}}_{i}^{t}}{{\tilde{y}}_{i}^{s}}\right) \end{array}$$

By minimizing $L_{\mathrm{soft}}$, we can train a student model to learn the predictions of the teacher model.

### 3.5. Learn Predictions from a Downstream Task

To better help our student model solve downstream tasks, we also define an objective function for our student model with the ground truths in each task. For each input instance $x_{i}$ in a task, we can obtain the output probability ${\tilde{y}}_{i}^{s}$ of a student model $f^{s}\left(x_{i};\theta_{s}\right)$ from the output logits $z_{i}^{s}$:

(16) $$\begin{array}{ccc} \;z_{i}^{s}=f^{s}\left(x_{i};\theta_{s}\right) & & \end{array}$$

(17) $$\begin{array}{cc} {\tilde{y}}_{i}^{s} & =\mathrm{softmax}(z_{i}^{s}) \end{array}$$

where $z_{i}^{s}$ represents the output logits of the student model. We define the objective with cross-entropy:

(18) $$\begin{array}{c} L_{\mathrm{hard}}=-\sum_{i=1}^{K}y_{i}\log{\tilde{y}}_{i}^{s} \end{array}$$

### 3.6. Distillation Objective

Finally, we combine all the objective functions for our overall LAD framework:

(19) $$\begin{array}{c} L_{LAD}=\alpha L_{\mathrm{soft}}+\beta L_{\mathrm{hard}}+\gamma L_{\mathrm{hidden}} \end{array}$$

where α, β, and γ are hyper-parameters controlling the importance of each loss function. In all the experiments, we set β equal to 1−α.

## 4. Experiments

### 4.1. Datasets

The General Language Understanding Evaluation (GLUE) [20] is a benchmark used to train and evaluate NLP models. This comprises nine datasets of natural language understanding (NLU) with either single-sentence or paired-sentence tasks. In this work, we use tasks from the GLUE benchmark to compare our proposed method with other task-specific distillation models. The tasks are described as follows:

#### 4.1.1. SST-2

The Stanford Sentiment Treebank [21] is a single-sentence sentiment classification task consisting of sentences extracted from movie reviews. Given an input sentence, the model has to determine whether the sentiment behind the statement is *Positive* or *Negative*.

#### 4.1.2. MRPC

The Microsoft Research Paraphrase Corpus [22] is a corpus consisting of sentence pairs collected from online news sources. Each sentence pair is labeled with human annotation, indicating whether two sentences are semantically equivalent.

#### 4.1.3. QQP

The Quora Question Pairs (https://quoradata.quora.com/First-Quora-Dataset-Release-Question-Pairs accessed on 22 January 2023) dataset contains about 300,000 question pairs collected from the Quora community’s question-answering website. This is a binary classification task where a model has to predict whether a pair of questions are semantically equivalent.

#### 4.1.4. MNLI

The Multi-Genre Natural Language Inference Corpus is a large-scale textual entailment dataset containing 393K training sentence pairs. Given a premise sentence P and a hypothesis sentence H, a model has to identify whether the premise entails the hypothesis, contradicts the hypothesis, or neither. There are two validation datasets: MNLI (matched) and MNLI (mismatched). The data sources for the premise sentences in the MNLI (matched) are the same as those in the training dataset.

#### 4.1.5. QNLI

The Question-answering NLI (QNLI) is a sentence pair classification task in which a model has to determine whether the sentence context contains the answer to the question. The question-context pairs are transformed from the Stanford Question Answering Dataset.

#### 4.1.6. RTE

The Recognizing Textual Entailment datasets combine a series of annual textual entailment challenges, including RTE1 [23], RTE2 [24], RTE3 [25], and RTE5 [26]. The sentences are from news and Wikipedia texts, and all datasets are converted into a two-class setting. Assume we are given a sentence pair. The RTE’s tasks is to let a model determine whether the first sentence entails the second sentence.

### 4.2. Teacher Model

We obtained the pre-trained model weights of the BERT_BASE_ encoder from HuggingFace’s model hub (https://huggingface.co/models accessed on 1 November 2022).To generate the task-specific teacher models, we followed the fine-tuning procedures provided by the original paper [1] and fine-tuned the model on each downstream task with the hyperparameters suggested by HuggingFace (https://github.com/huggingface/transformers/tree/main/examples/pytorch/text-classification accessed on 1 November 2022). We then used each task-specific teacher model to generate the internal hidden states and the soft labels [8].

### 4.3. Baselines and Implementation Details

We summarize the existing methods of knowledge distillation in Table 1. PKD [14], BERT-of-Theseus [27], and ALP-KD [15] are used as the baseline models in our experiments due to their direct relatedness to our work in task-specific distillation. Task-agnostic methods such as DistilBERT [10] and MobileBERT [12] are not directly comparable to ours since they use a pre-training compression setting instead of performing distillation during training for downstream tasks.

Our proposed student models, LAD_6_ and LAD_4_, are lightweight versions of BERT, where the subscript indicates the number of Transformer layers [28] in the model. Each Transformer layer contains 12 attention heads, with a hidden dimension size of 768. Before training, each *m*-th layer in the student models was initialized with the weights of the corresponding *n*-th layer in a pre-trained BERT_BASE_, where *n* is equal to *m* multiplied by *p*, and *p* is the number of teacher layers divided by the number of student layers. For example, the first layer of LAD_6_ is initialized with the parameters from the second layer of the pre-trained teacher model; the first layer of LAD_4_ is initialized with the parameters from the third layer of the pre-trained teacher model. After the initialization of student models, we created the Gate Network by stacking gate blocks. The number of gate blocks equals the number of teacher layers. It should be noted that there is no parameter-sharing between each gate block, and the weight matrix of each gate block is initialized with Xavier Initialization [29].

During the f LAD_6_ and LAD_4_ training, the batch size and sequence length were 32 and 128 across all tasks, respectively. In addition, we used different AdamW optimizers [30] to update the Gate Network and the student model separately. For our LAD_6_ student, the training epochs on SST-2, MRPC, QQP, MNLI, QNLI, and RTE are 20, 20, 5, 4, 4, and 10, respectively. For LAD_4_, the training epochs on the same six tasks were 20, 30, 5, 4, 10, and 30. For the other hyperparameters, we set the softmax temperature τ from {5, 10, 20}, soft target weight α from {0.2, 0.5, 0.7}, and the aggregated hidden loss weight γ from {100, 500, 1000}. The learning rate of student models was {$1\times 10^{-4}$, $3\times 10^{-4}$, $5\times 10^{-4}$, $7\times 10^{-4}$} for all the GLUE tasks.For the Gate Network, the learning rates for the QQP task and the RTE task were {$1\times 10^{-7}$, $3\times 10^{-7}$, $5\times 10^{-7}$} and {$1\times 10^{-5}$, $3\times 10^{-5}$, $5\times 10^{-5}$} respectively, and the learning rates for the remaining tasks were {$1\times 10^{-6}$, $3\times 10^{-6}$, $5\times 10^{-6}$} (Learning rate warmup over the first 10% training steps for RTE and MRPC, 30% training steps for the remaining tasks.). Then, we performed a grid search over τ, α, γ, and learning rates mentioned above to choose the best model.

## 5. Results

### 5.1. Results on GLUE Test Sets

We evaluated our six-layer LAD student model using the GLUE test sets and summarized the results in Table 2. The results show that our approach outperforms PKD [14] and BERT-of-Theseus [27] in most of the GLUE tasks reported in Table 2, except RTE. However, due to the small data size of RTE, the difference (0.4%) between LAD and BERT-of-Theseus is marginal. Furthermore, the proposed LAD framework obtained a much higher MNLI score than the other two baselines. When directly compared with PKD, LAD performs better in all of the tasks. PKD even exhibited a massive decrease in MRPC when the model was evaluated in terms of accuracy. These results show that the proposed LAD approach can more effectively distill the knowledge of the teacher model than PKD.

### 5.2. Results on GLUE Development Sets

We evaluated our approach with the six-layer and four-layer student models and compared the results with ALP-KD [15] and BERT-of-Theseus [27] on the GLUE development sets. Both six-layer and four-layer LAD students outperform the baselines in nearly all of the GLUE tasks, as reported in Table 3. These results show that our method is more robust than the other two methods, and we found that LAD significantly outperforms ALP-KD on larger datasets, such as QQP and MNLI. In addition, although BERT-of-Theseus performs well with six-layer student models, it shows dramatic decreases in performance when the model size becomes smaller.

### 5.3. Comparison with the Attention Mechanism

**Figure 2:**
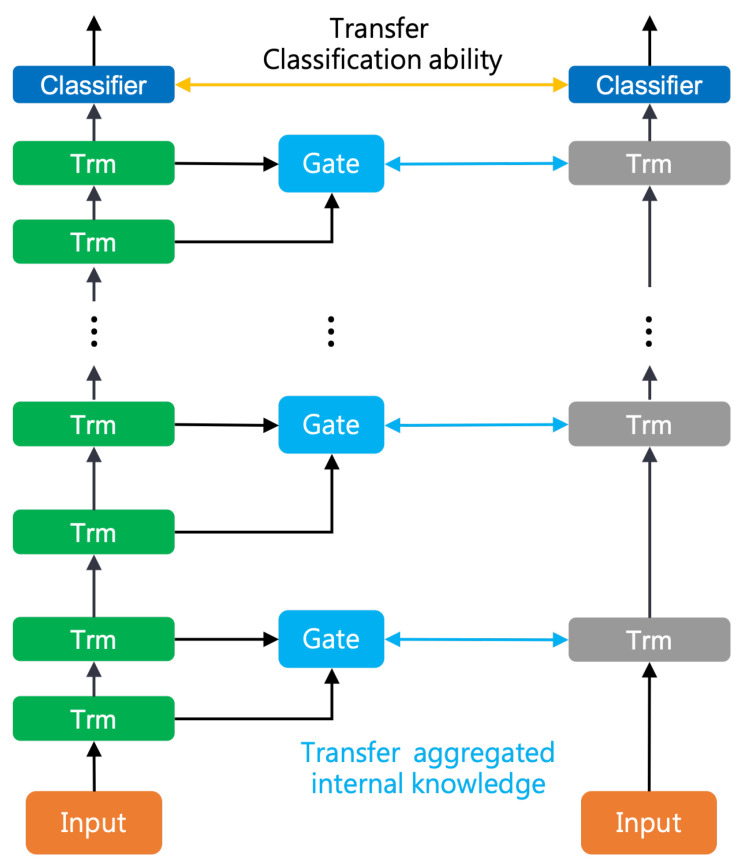
The model structure of LAD-NO. The left shows the teacher model, and the right indicates the student model.

We demonstrated that our LAD students perform better on GLUE tasks than ALP-KD students [15] in Table 3. As the ALP-KD framework is close to our approach, in this section, we further investigate the difference between LAD and ALP-KD. However, directly comparing the two approaches is not intuitive due to the different framework structures, which we show in Figure 1. Therefore, we intend to discover whether the gate block mechanism is better than the attention mechanism. Passban et al. (2020) built a competitive baseline called ALP-NO, which only applied attention between every *p* teacher layer. To directly compare our approach with the attention mechanism, we constructed an LAD-NO model, which is similar to ALP-NO, with the gate blocks in our LAD framework. We show the LAD-NO structure in Figure 2.

We list the LAD-NO and ALP-NO scores for the GLUE development sets in Table 4. For the experiments of the six-layer student models, we observe that LAD-NO_6_ outperforms ALP-NO_6_ on four of the six GLUE tasks. These results show that our approach works better than the attention mechanism from internal knowledge distillation in most of the cases.

### 5.4. Analysis of the Directions of Gates

In this section, we discuss the importance of the sentence processing order in the BERT model [1,16]. The Gate Network in our LAD framework is designed to propagate the distilled knowledge from lower hidden layers to higher ones (Figure 1c). We want to know if reversing the order of the Gate Network affects the performance of LAD student models due to the sentence processing nature of BERT. Thus, we aimed to propagate the distilled knowledge from higher hidden layers to lower ones, and reported the results in Table 5. Comparing LAD_6_ with LAD_6_-*Reverse*, we found that, once we reverse the order of our Gate Network, the performance decreases. The result also implies that the design of our layer-wise distillation framework can benefit model performance on downstream tasks.

### 5.5. Analysis of Aggregated Knowledge

In our proposed method, the Gate Network is the critical component when aggregating knowledge from multiple layers of the teacher model. This section investigates how much aggregated knowledge and how many gate blocks we need for more effective distillation. We conducted experiments with six different LAD_6_ models on three of the GLUE development sets. Each student model learns the different extent of aggregated knowledge produced by the different number of gate blocks. We summarize the results of this experiment in Table 6. According to the experimental results, the more aggregated knowledge the students learn, the better the performances the students will achieve. This observation implies that learning more aggregated knowledge improves distillation, which also explains the effectiveness of layer-wise distillation.

## 6. Conclusions

Model compression for large-scale pre-trained language models is imperative in the current trend of natural language processing when utilizing them in real-world applications. This work proposes a novel task-specific layer-wise distillation framework to leverage knowledge in the teacher model without manually skipping any teacher layers, while retaining the sentence processing nature of BERT. Our experiments demonstrate that the proposed method outperforms the baseline approaches on most GLUE tasks and shows the effectiveness of our LAD framework. Furthermore, the proposed method provides an improved solution for model compression, which can be further applied to IoT or edge devices for better deployment of BERT for natural language applications.

## Figures and Tables

**Table 1:** Comparison with previous knowledge distillation approaches for transformer-based models.

Method	Teacher Model	Use of External Data?	Knowledge Distillation Type
BERT_BASE_ [1]	-	-	-
BERT-PKD [14]	BERT_BASE_	No	Task-specific
BERT-of-Theseus [27]			
ALP-KD [15]			
DistilBERT [10]	BERT_BASE_	Yes	Task-agnostic
MobileBERT [12]	IB-BERT_LARGE_	Yes	
TinyBERT [11]	BERT_BASE_	Yes	
MINILM [13]	BERT_BASE_	No	
BERT-EMD [18]	BERT_BASE_	Yes	
LAD (ours)	BERT_BASE_	No	Task-specific

**Table 2:** Results of the six-layer student models from the GLUE test server. Two evaluation metrics with a slash (F1/accuracy scores) are reported for QQP, and accuracy scores are reported for the other tasks.

Model	#Params	SST-2	MRPC	QQP	MNLI m/mm	QNLI	RTE
BERT_BASE_ * [1]	110M	93.2	89.1	71.7/89.2	84.2/84.3	91.0	67.4
BERT-PKD [14]	66.5M	92.0	85.0	70.7/88.9	81.5/81.0	89.0	65.6
BERT-of-Theseus [27]	66.5M	92.2	87.6	71.6/89.3	82.4/82.1	89.6	66.2
LAD	66.5M	92.5	87.6	72.2/89.4	84.0/83.0	90.1	65.8

* Teacher model for LAD.

**Table 3:** Results of six-layer and four-layer student models from the GLUE development sets. Two evaluation metrics with a slash (F1/accuracy scores) are reported for MRPC and QQP, and accuracy scores are reported for the other tasks. Xu et al. [27] averaged the scores of MNLI-m and MNLI-mm.

Model	#Params	SST-2	MRPC	QQP	MNLI m/mm	QNLI	RTE
BERT_BASE_ * [1]	110M	93.46	90.81/87.01	88.02/91.07	84.6/85.04	91.94	70.04
BERT-of-Theseus [27]	66.5M	91.5	89.0/–	89.6/–	82.3	89.5	68.2
ALP-KD_6_ [15]	66.5M	91.86	–/85.05	–/90.73	81.86/–	89.67	68.59
LAD_6_	66.5M	91.86	89.59/84.56	88.20/91.16	83.78/84.40	90.74	68.59
BERT-of-Theseus [27]	52.5M	89.1	87.5/–	88.7/–	80.0	86.1	61.9
ALP-KD_4_ [15]	52.5M	90.37	–/82.57	–/90.54	79.62/–	87.02	67.15
LAD_4_	52.5M	91.74	88.71/83.09	87.56/90.78	81.01/81.47	89.24	67.15

* Teacher model for LAD.

**Table 4:** Performance comparisons between LAD-NO and ALP-NO [15] on GLUE development sets. The value in parentheses represents the difference in performance between the student model and its corresponding teacher model.

Model	#Params	SST-2	MRPC	QQP	MNLI m/mm	QNLI	RTE
BERT_BASE_ * [1]	110M	93.46	90.81/87.01	88.02/91.07	84.6/85.04	91.94	70.04
ALP-NO_6_ ^‡^	66.5M	91.86	–/85.78	–/90.64	81.99/–	89.71	68.95
LAD-NO_6_	66.5M	92.32	88.52/82.84	87.59/90.77	83.42/83.66	90.72	68.95

^‡^ Results from [15]; * Implemented by ourselves.

**Table 5:** Analysis of the propagation direction of the Gate Network. LAD_6_-*Reverse* indicates that we reversed the direction in the Gate Network during knowledge distillation.

Prediction Set	Strategy	SST-2	MRPC	QQP	MNLI (m/mm)	QNLI	RTE
GLUE test	LAD	92.5	87.6/82.0	72.2/89.4	84.0/83.0	90.1	65.8
	LAD-*Reverse*	91.4	84.3/75.9	71.9/89.1	83.8/82.9	89.9	63.1

**Table 6:** Performance comparisons of transferring different extent of aggregated internal knowledge.

Gate ID	Student Layer ID	SST-2	QNLI	RTE
12	6	87.04	85.92	55.60
12,10	6,5	87.73	86.84	57.04
12,10,8	6,5,4	90.25	88.32	58.84
12,10,8,6	6,5,4,3	90.48	89.27	59.21
12,10,8,6,4	6,5,4,3,2	91.28	90.43	66.43
12,10,8,6,4,2	6,5,4,3,2,1	91.86	90.76	67.87

## Data Availability

Data used in the reported results can be found at the website of the GLUE benchmark https://gluebenchmark.com/tasks (accessed on 1 November 2022). Our code is available at https://github.com/IKMLab/LAD (accessed on 22 January 2023).

## Abbreviations

The following abbreviations are used in this manuscript:

LAD	Layer-wise Adaptive Distillation
BERT	Bidirectional Encoder Representations from Transformers
KD	Knowledge Distillation
NLI	Natural Language Inference
GLUE	The General Language Understanding Evaluation benchmark
SST	The Stanford Sentiment Treebank
MRPC	Microsoft Research Paraphrase Corpus
QQP	Quora Question Pairs
MNLI	Multi-Genre Natural Language Inference
QNLI	Question-answering NLI
RTE	Recognizing Textual Entailment
